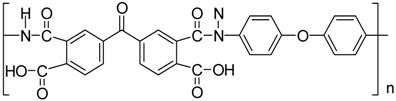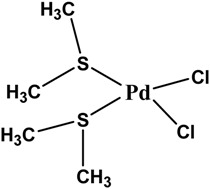# Polymer Nanocomposite Film with Metal Rich Surface Prepared by In Situ Single-Step Formation of Palladium Nanoparticles: An Interesting Way to Combine Specific Functional Properties

**DOI:** 10.3390/nano6100188

**Published:** 2016-10-18

**Authors:** David Thompson, David Kranbuehl, Eliane Espuche

**Affiliations:** 1 Departments of Chemistry and Applied Science, College of William and Mary, Williamsburg, VA 23187, USA; dwthom@wm.edu (D.T.); dekran@wm.edu (D.K.); 2 University of Lyon, University Lyon1, IMP (Ingénierie des Matériaux Polymères) UMR CNRS 5223, 69622 Villeurbanne, France

**Keywords:** metal nanoparticles, polyimide films, optical properties, gas barrier material, hydrogen trap material

## Abstract

This paper presents a continuous single-step route that permits preparation of a thermostable polymer/metal nanocomposite film and to combine different functional properties in a unique material. More precisely, palladium nanoparticles are in situ generated in a polyimide matrix thanks to a designed curing cycle which is applied to a polyamic acid/metal precursor solution cast on a glass plate. A metal-rich surface layer which is strongly bonded to the bulk film is formed in addition to homogeneously dispersed metal nanoparticles. This specific morphology leads to obtaining an optically reflective film. The metal nanoparticles act as gas diffusion barriers for helium, oxygen, and carbon dioxide; they induce a tortuosity effect which allows dividing the gas permeation coefficients by a factor near to 2 with respect to the neat polyimide matrix. Moreover, the ability of the in situ synthesized palladium nanoparticles to entrap hydrogen is evidenced. The nanocomposite film properties can be modulated as a function of the location of the film metal-rich surface with respect to the hydrogen feed. The synthesized nanocomposite could represent a major interest for a wide variety of applications, from specific coatings for aerospace or automotive industry, to catalysis applications or sensors.

## 1. Introduction

The combination of polymers with metal particles is of great interest for a large range of applications going from sensors to catalysis, barrier applications, antimicrobial applications, conductive materials, or reflective materials [1,2,3,4,5]. The efficiency of the materials facing the intended applications depends both on the metal nature and content and on the metal particles dispersion state and location. The presence of metal nanoparticles in a sufficient amount at the material surface should theoretically obtain high reflectivity, bactericidal properties, or barrier properties for example. Surface metallization of various substrates has been then accomplished and chemical, physical or sputtering deposition techniques have been widely used [6,7]. However, such surface treatments are a supplementary step in the material preparation. Moreover, the lack of adhesion between the polymer substrates and the deposits as well as the differences in mechanical properties between polymers and inorganic coatings are considered to be the weak points of these multilayer materials. They can be at the origin of dramatic problems especially for flexible samples such as thin films or for maintaining functional properties over time [7,8]. Several works reported in the literature have described alternative ways to obtain thin deposits made of silver on various polymer substrates. In these works, silver salts have been used as precursors of silver nanoparticles and different routes have been developed to obtain the metal nanoparticles and the intended specific location of these nanoparticles [9,10,11,12]. As examples, the formation of a silver surface layer has been obtained by a photochemical reaction performed on a solution of the silver precursor deposited on the polymer substrate [10]. More interestingly, nanocomposite materials with specific morphologies have been achieved from a single polymer/silver salt solution [11,12]. In that case, a precursor film composed of the polymer and of the silver salt is generally prepared and then, metal nanoparticles are in situ formed. Depending on the polymer/silver salt system used, the formation of the silver nanoparticles is either obtained by swelling the film in a solution containing a chemical reducing agent (very often, sodium borohydrate) [11] or by a specific post-curing step [12]. The last route appears as particularly interesting as it allows forming the multi-material in a single continuous step. It also avoids the use of chemical reducing agents which are not environmentally friendly. Although this route has shown its interest for silver nanoparticles, it has not been extended to other metal types with the aim of preparing multifunctional materials.

In this paper, we will show that a continuous single-step route can be used to prepare polymer/metal nanocomposite films which combine different functional properties. In this work, palladium nanoparticles are formed in situ in a thermostable polymer matrix thanks to a thermal treatment. The resulting nanocomposite film is composed of a metal-rich surface and of a homogeneous dispersion of nanoparticles in the bulk. It is characterized by a high thermal stability and the impact of the morphology on the functional properties is studied. Thanks to the presence of a significant amount of palladium nanoparticles at the film surface, a high reflectivity is provided. An interesting increase of barrier properties is achieved for a large variety of gases. Finally, the specific nature and location of the in situ formed nanoparticles also permit to modulate hydrogen transport properties. Thus different functional properties are combined in a single nanocomposite material which could be used in a wide variety of applications, going from specific coatings for aerospace or automotive, to catalysis applications or sensors.

## 2. Results

### 2.1. Morphology and Structure of the Films

**Figure 1:**
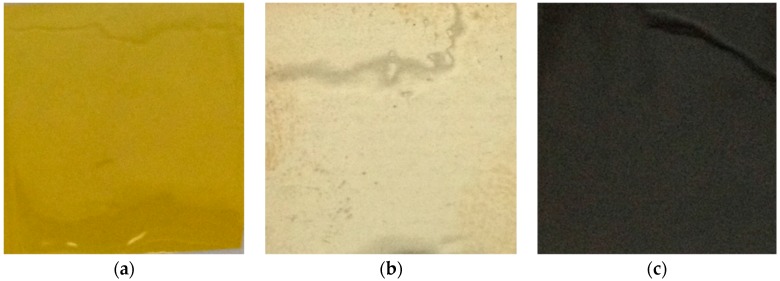
Observation of the studied films at the macroscale: (**a**) neat polyimide film; (**b**) nanocomposite film surface formed in contact with air; and (**c**) nanocomposite film surface formed in contact with the glass plate.

The neat film was transparent and exhibited the typical yellow color observed for polyimide materials (Figure 1a). The nanocomposite film was opaque and clear differences were observed between the film surface which was in contact with air during the film formation and the film surface which was in contact with the glass plate. A reflective metallized homogeneous surface was observed in the first case (Figure 1b) whereas a dark colored homogeneous surface was observed in the second one (Figure 1c).

**Figure 2:**
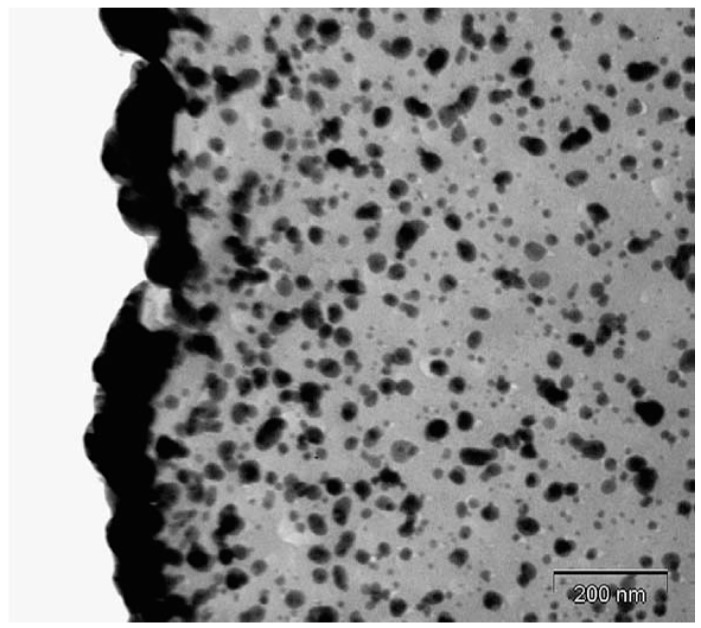
Transmission electron microscopy (TEM) image of the cross section of the nanocomposite film (scale bar: 200 nm).

Transmission electron microscopy (TEM) analysis performed on the cross section of the nanocomposite film demonstrated the presence of a homogeneous dispersion of nanometer sized particles within the bulk material. The size of the in situ formed nanoparticle was around 10 nm. Densely packed nanoparticles were also clearly observed at the film surface formed in contact with air. The mean thickness of the nanoparticle-rich surface layer was around 60 nm (Figure 2).

**Figure 3:**
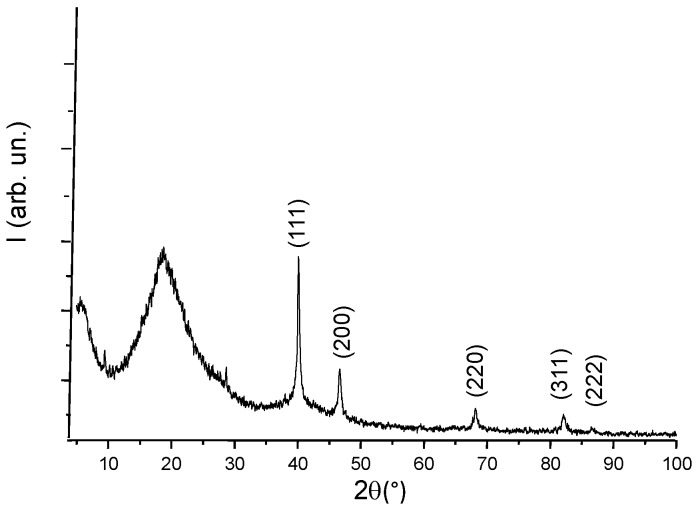
X-ray diffraction (XRD) pattern of the nanocomposite film: the diffraction rays of the metal nanoparticles are pointed and identified in the XRD pattern.

The structure of the nanocomposite film was studied thanks to X-ray diffraction (XRD) analysis. XRD pattern exhibited a halo in the range of 2θ below 35° which is the contribution of the amorphous polymer matrix (Figure 3). The structure of the formed nanoparticles was identified thanks to their characteristic diffraction peaks. The diffraction peaks of f.c.c. metallic palladium (namely (111), (200), (220), (311), and (222)) were clearly observed in Figure 3.

The differential scanning calorimetry analysis performed on the neat polymer film revealed only a change in heat capacity (*∆C_p_*) which allowed determination of the polymer glass transition temperature (T_g_ = 272 °C). Any endothermic peak was observed on the differential scanning calorimetry (DSC) traces in agreement with XRD results and the amorphous state of the polymer. The DSC scan performed on the nanocomposite film did not show any *∆C_p_* change in the temperature range up to 300 °C. This result tends to show that the polymer chain mobility was restricted in the nanocomposite film with respect to neat polymer film [13].

### 2.2. Gas Transport Properties

**Figure 4:**
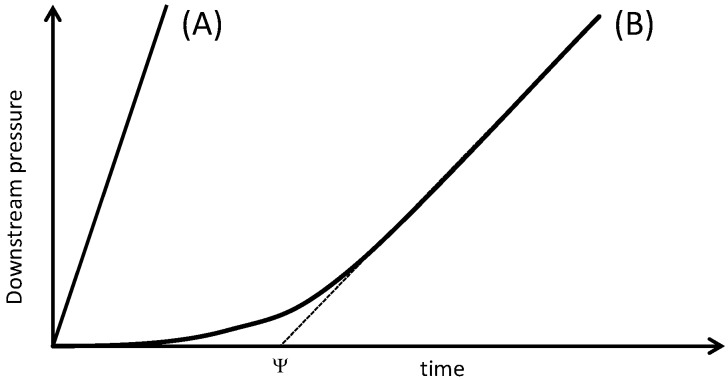
Typical gas permeation curves observed for the neat polymer film.

The typical shapes of the curves representing the evolution of the downstream pressure as a function of time observed during the permeation experiments performed on the neat polymer film are shown in Figure 4.

Type A curve did not allow determination of a time lag value (ψ) and was obtained for the gases with small kinetic diameter (helium and hydrogen) due to their very fast diffusion rate and the small thickness of the polymer sample. Type B curve was observed for oxygen and carbon dioxide and permitted to calculate both permeability and diffusion coefficients [14]. The transport properties of the neat polyimide film are summarized in Table 1. The measured permeation properties were in good agreement with those reported in the literature for the same polymer matrix films [15].

The gas permeation properties were measured in the same conditions for the nanocomposite film and the relative permeability coefficient and relative apparent diffusion coefficient (e.g., the ratio between the transport coefficients measured on the nanocomposite film and the same ones measured on the neat polymer film) were determined. The permeation experiments were performed for two different configurations: Either the metallized film surface was placed at the upstream side in the cell and directly faced the feed gas or it was placed at the downstream side, meaning that gases should first diffuse through the bulk of the nanocomposite film before reaching the metal rich film surface. The relative permeability and diffusion values obtained for He, O_2_, and CO_2_ are summarized in Table 2.

For all these gases, the permeation curve type did not change going from the neat polymer film to the nanocomposite film. The relative permeability values were lower than 1, meaning that the presence of the metal nanoparticles led to a decrease in the permeation flux through the material. This behavior was mainly due to a decrease of the gas diffusion rate. Indeed, the relative diffusion and permeation values were similar. It is noteworthy that the extent of the permeability decrease was similar for all gases (close to a factor 2). It could at last be observed that the position of the film metal-rich surface in the permeation cell did not lead to significant differences in the transport properties, meaning that the permeation curves were superimposed whether the metallized surface of the sample was located in the upstream or downstream side of the cell.

**Figure 5:**
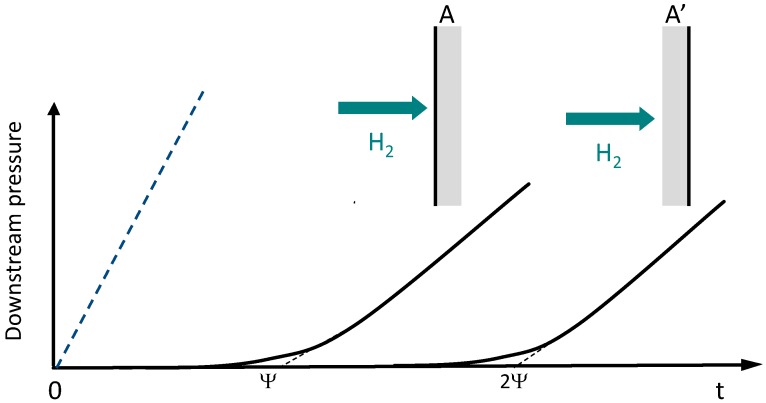
Schematic representation of the hydrogen permeation curves obtained for the neat polymer film (dotted line) and the nanocomposite sample (continuous line). The permeation curves of the nanocomposite are represented for two configurations: case A: the metal-rich surface is placed at the upstream side in the cell- case A’: the metal-rich surface is placed at the downstream side in the cell.

Hydrogen behaved in a totally different way with respect to He, O_2_, and CO_2_. Figure 5 shows that, contrary to the neat polymer film, the hydrogen permeation curves of the nanocomposite film exhibited a significant time lag. Moreover, the time lag value increased by a factor 2 when the film metal-rich surface was located at the downstream side of the cell (Case A’ in Figure 5 in comparison with case A). The apparent diffusion coefficient value determined from the time lag data was 4.08 × 10^−13^ m^2^·s^−1^ when the sample metallized surface was placed at the upstream side of the cell and 1.92 × 10^−13^ m^2^·s^−1^ when it was located at the downstream side. The permeability coefficient values were equal to 5.92 × 10^−16^ mol·m·m^−2^·s^−1^·Pa^−1^ and 6.56 × 10^−16^ mol·m·m^−2^·s^−1^·Pa^−1^, respectively. They were thus very similar and two times lower than the permeability coefficient measured on the neat polymer film. All these results underlined a modification of the gas transport mechanism going from neat matrix to the nanocomposite material.

## 3. Discussion

Thermal curing step-up to 300 °C of the homogeneous (polyamic acid) polymer/metal precursor system led to reduction of the metal ions and metal particle formation with cycloimidization of the amic acid. Crystalline spherical metal nanoparticles were formed in situ within the amorphous polymer matrix. In addition, a high concentration of metal nanoparticles was obtained at the film external face in contact with air during the film formation, allowing highly reflective nanocomposite materials to be obtained. This metal-rich surface layer was highly cohesive due to in situ synthesis of metal particles from metal precursor embedded in the polymer matrix. Moreover, despite the low content of metal nanoparticles contained within the nanocomposite film (5 wt %), it could be concluded from DSC analysis that a significant reduction of polymer chain mobility occurred in this material as *∆C_p_* variation was too low to be detected or shifted to a temperature higher than 300 °C, the first case being more likely to occur.

The gas transport properties measured for He, O_2_, and CO_2_ highlighted the significant barrier effect brought by the metal nanofillers. The high decrease of gas permeability could be assigned to a tortuosity effect created by the nanofillers [16]. The combination of the homogneous dispersion of the nanofillers within the bulk polymer matrix and of densely packed nanoparticles at one film surface allowed dividing the permeability coefficient by a factor 2 for a low total amount of nanoparticles (5 wt % corresponding to less than 1 vol %). As a comparison, according to Maxwell law [17], the theoretical permeability decrease should be less than 1% considering a homogeneous dispersion of the same total content of spherical impermeable nanoparticles. From our gas transport data, it was also checked that the tortuosity effect involved in the gas transport mechanism did not depend on the position of the sample metal-rich surface in the permeation cell. 

The decrease of the hydrogen permeability coefficient observed for the nanocomposite was of the same order as the one observed for the other gases. Moreover, the obtained permeability value did not depend on the position of the film metal-rich surface within the cell. It could thus be concluded that the hydrogen transport properties measured in the steady state were governed, as for the other studied gases, by the tortuosity effect created by the metal nanoparticles. However, the tortuosity effect could not explain the presence of the significant time lag observed in the hydrogen permeation curve of the nanocomposite and the variation of the time lag value evidenced as a function of the location of the metal-rich surface in the permeation cell. In agreement with previous literature data [18,19], the presence of a significant time lag could be assigned to the entrapment of hydrogen species by the palladium nanoparticles. One major and significant result obtained in the present work is to show that, for materials containing non-homogeneously dispersed nanoparticles, the time lag value could highly depend on the nanoparticle location with respect to hydrogen feed. The membrane behavior observed for the nanocomposite sample is then comparable to the behavior reported by some authors in the case of so-called reactive membranes [20]. From the data obtained in this work, further finite element modeling will be now developed and extended to predict the functional properties of membranes with different morphologies, metal contents, and different polymer matrix properties with the aim to design the best materials as a function of targeted functional applications.

## 4. Materials and Methods

### 4.1. Chemicals

3,3’,4,4’-benzophenone tetracarboxylic dianhydride (BTDA) from Chriskev Company (Lenexa, KS, USA), Inc. was vacuum dried at 150 °C for 5 h before use. 4,4’-diaminodiphenylether (ODA) was used as received from Chriskev Company (Lenexa, KS, USA), Inc. Palladium (II) chloride was purchased from Pfaltz Bauer (Waterbury, CT, USA), Inc. Dimethyl acetamide (DMAC) and dimethylsulfide were purchased from Aldrich Chemical (St. Louis, MO, USA).

### 4.2. Preparation of the Metal Rich Surface Nanocomposites

The poly(amic acid) polymer was prepared from the reaction of ODA with BTDA. The diamine was first dissolved in DMAC (at 12% w/w) in a resin kettle flushed with dry nitrogen and BTDA was then added (offset at 1 mol % relative to ODA). The reaction was led during 6 h. Bis(dimethyl sulfide) dichloro palladium was obtained by dissolving palladium chloride in DMAC and adding 4 mole equivalent of dimethylsulfide. The chemical structures of the polymer and metal precursor are presented in Table 3.

To prepare the nanocomposite films, the polyamic acid solution (15 wt % polymer in DMAC) was mixed with the metal complex solution and vigorously stirred at room temperature to obtain a homogeneous, clear solution. The polymer to metal precursor mole ratio was fixed in order to obtain a palladium weight amount of 5%. The solution was cast on a glass plate and then subjected to a slowly flowing dry air for 15 h at room temperature. It was then heated to 135 °C at a heating rate of 5.5 °C/min, held at 135 °C for 1 h, and then heated to 300 °C at a heating rate of 1 °C/min. The last heating step was an isotherm of 1 h at 300 °C before slow cooling to room temperature. Reference polymer films were prepared from the neat polymer solution according to the same curing cycle as that used for the nanocomposite film. The obtained films had a thickness of 40 μm.

### 4.3. Characterization

The location and size of the in situ synthesized nanoparticles were observed by transmission electron microscopy (Philips CM120 transmission electron microscope, Amsterdam, The Netherlands), with an accelerating potential of 80 kV. Samples were microtomed at room temperature with a Leica EMFCS (Heerbrugg, Switzerland) instrument equipped with a diamond knife to obtain slices of about 80 nm thickness. The structure of the nanoparticles was examined using wide angle X-ray diffraction (XRD, Siemens D500) (Bruker, Billerica, MA, USA)with Cu Kα of 0.1542 nm radiation. The glass transition temperature of the polymer matrix was determined by Differential Scanning Calorimetry (TA Instruments 2920 apparatus, New Castle, DE, USA). DSC heating scans were performed on 10 mg samples at a heating scan of 10 °C/min in the temperature range from room temperature to 300 °C.

Gas permeation properties were determined at 20 °C under an upstream pressure of 3 bar. A high vacuum desorption step was performed prior to each experiment. The gas of interest (namely, oxygen, carbon dioxide, helium, and hydrogen) with a purity level above 99.98% was introduced in the upstream compartment of the cell and the pressure in the downstream compartment was followed as a function of time with a 10 Torr datametrics pressure sensor. The gas transport coefficients were calculated considering that a Fickian mechanism governed the transport properties [14]: the permeability coefficient was determined from the data obtained in steady state and expressed in mol·m·m^−2^·s^−1^·Pa^−1^; the time to reach the steady state, defined as the time lag (ψ) was also determined and it allowed calculation of an apparent diffusion coefficient *Da*.

*Da* = *l*^2^/6ψ, (1)

where *l* is the membrane thickness. *Da* values were expressed in m^2^·s^−1^.

Permeation experiments were performed either with the metal rich surface of the film placed on the upstream or on the downstream side of the cell. Permeation data were the average of three measurements performed on three different samples. The reproducibility was better than 5%.

## 5. Conclusions

This paper presents a single continuous step route that allows preparing polymer/metal nanocomposite films which combine different functional properties. In this work, palladium nanoparticles have been formed in situ in a thermostable polymer matrix thanks to a defined thermal treatment and a nanocomposite film with one metal-rich surface which has been obtained. The interest of this specific surface layer is first to provide a high reflectivity to the material. It also creates an interesting increase of barrier properties for a large variety of gases. At last, the specific nature and location of the in situ formed nanoparticles has permitted preparation of materials with modulated gas transport properties towards hydrogen. This type of nanocomposites could present a high interest for a wide variety of applications, going from specific coatings for aerospace or automotive industry, to catalysis applications or sensors.

## Figures and Tables

**Table 1:** Gas permeation properties of the neat polymer film.

Gas	Permeability Coefficient (10^−16^ mol·m·m^−2^·s^−1^·Pa^−1^)	Apparent Diffusion Coefficient (10^−14^ m^2^·s^−1^)
He	15.00	/ ^1^
H_2_	11.75	/ ^1^
O_2_	0.50	22.7
CO_2_	1.87	4.7

^1^ could not be determined.

**Table 2:** Helium, oxygen, and carbon dioxide permeation relative properties of the nanocomposite film as a function of the location of the sample metal-rich surface layer.

Gas	Location of the Metallized Surface	Relative Permeability	Relative Diffusion
He	Upstream	0.62 ± 0.06	/ ^1^
	Downstream	0.59 ± 0.06	/ ^1^
O_2_	Upstream	0.47 ± 0.05	0.55 ± 0.06
	Downstream	0.49 ± 0.05	0.55 ± 0.06
CO_2_	Upstream	0.46 ± 0.05	0.53 ± 0.05
	Downstream	0.47 ± 0.05	0.54 ± 0.05

^1^ could not be determined due to type A permeation curves.

**Table 3:** Chemical structure of the components used as base materials for the nanocomposite preparation.

Poly(Amic Acid) Polymer	Palladium Precursor